# Extramedullary Hematopoiesis Presenting as Pleural Thickening in a Patient With Choroidal Melanoma

**DOI:** 10.7759/cureus.81428

**Published:** 2025-03-29

**Authors:** Shuai Li, Anusha Mubin, David Cantu

**Affiliations:** 1 Pathology, Creighton University School of Medicine, Omaha, USA

**Keywords:** extramedullary hematopoiesis, hematopathology, pathology, pleural thickening, polycythemia vera

## Abstract

Extramedullary hematopoiesis (EMH), the generation of blood cells by organs other than the bone marrow, most often occurs in the spleen or liver. However, it has been known to uncommonly occur in other locations. EMH in these locations does not consistently manifest in the same way, making this a difficult diagnosis to make. We report the case of a 79-year-old male former smoker with a history of choroidal melanoma and polycythemia vera who presented with a chronic cough and weight loss of 30 pounds over the last year. A CT scan revealed a 15 mm right basilar pleural enhancement suspicious for malignancy on imaging. Upon biopsy, it was found that the pleural enhancement was EMH due to post-polycythemia vera myelofibrosis. EMH can be difficult to distinguish from metastasis, especially in atypical locations and in patients with a history of malignancy.

## Introduction

Extramedullary hematopoiesis (EMH) is a compensatory process in which hematopoiesis occurs outside of the bone marrow, typically in response to stressors that disrupt normal marrow function [1]. Stressors can include infection, neoplasms, anemia, or metabolic stress [1]. Myeloproliferative neoplasms, such as primary myelofibrosis and polycythemia vera, are the most common underlying stressors [2,3].

EMH is typically found in the spleen or liver, possibly because hematopoietic progenitor cells are filtered through the spleen and liver and entrapped there [4]. However, in rare cases, EMH can occur in atypical locations, including the pleura, lungs, lymph nodes, paraspinal region, and skin [4]. The presence of EMH at such sites can pose significant diagnostic challenges, particularly in patients with a history of malignancy or hematologic disorders, as it may be mistaken for metastatic disease.

In this article, we discuss the case of a patient with a history of choroidal melanoma and polycythemia vera who presented with EMH as pleural thickening - an uncommon presentation that highlights the importance of recognizing EMH as a potential diagnostic consideration in patients with unusual findings.

## Case presentation

We report the case of a 79-year-old male patient with a history of polycythemia vera and choroidal melanoma who developed a chronic cough and significant weight loss. Imaging showed pleural enhancements concerning for malignancy, but a biopsy confirmed EMH, highlighting the diagnostic challenge in this atypical presentation.

The patient, a former smoker, presented with a persistent worsening cough for six months and an unintentional weight loss of 30 pounds over the past year. He also had a history of JAK2+ polycythemia vera (managed with baby aspirin, hydroxyurea, and as-needed phlebotomy) and a remote history of malignant choroidal melanoma in the left eye that was in remission. With the exception of decreased breath sounds in both lung bases, the physical exam was largely unremarkable, with no wheezes, rales, or rhonchi noted. Vitals were within the normal range.

**Figure 1:**
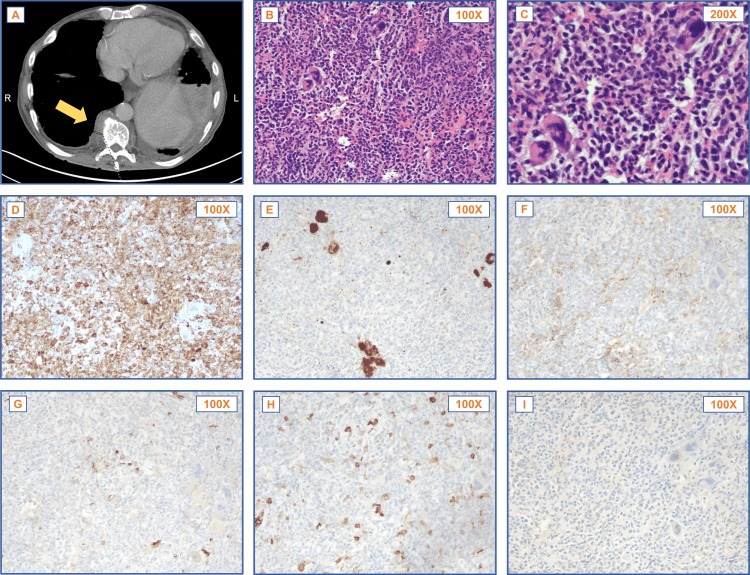
Imaging and histopathologic evaluation of pleural EMH Patient CT imaging (A) demonstrated suspicious pleural thickening, which did not exhibit fat deposition that could differentiate EMH from malignancy (arrow). H&E histology (B, C) and IHC stains for myeloperoxidase (D), CD61 (E), glycophorin A (F), CD34 (G), CD117 (H), and HMB-45 (I) highlighted three lineages of cells with a normal bone marrow-like distribution, which supported the diagnosis of EMH. EMH: extramedullary hematopoiesis; IHC: immunohistochemical; H&E: hematoxylin and eosin

A CT chest with contrast was performed after this visit, revealing enlarged left hilar and subcarinal lymph nodes, left lower lobe consolidation, and severe splenomegaly. Initially, the tentative diagnosis was pneumonia. However, the patient continued to experience unintentional weight loss, prompting a second CT scan of the chest, abdomen, and pelvis a month later. This scan showed a 15 mm right basilar pleural enhancement, a left pleural enhancement, and a possible left chest wall nodule (Figure 1A). Given the patient’s history of smoking, rapid weight loss, and melanoma, there were concerns that pleural enhancements indicated malignancy. Therefore, three weeks later, a bronchoscopy with endobronchial ultrasound was performed. The left lower lobe consolidation or the examined lymph nodes were not found to be malignant.

Two weeks after the bronchoscopy, the patient was referred for a biopsy of the pleural enhancements in both lungs. The left lung base biopsy showed inflammation and fibrinopurulent exudate, indicating bronchopneumonia. The histology of the right lung pleural enhancement biopsy revealed a diverse population of hematopoietic cells, suggesting active hematopoiesis at an extramedullary site (Figures 1B-1C). Immunohistochemical staining was instrumental in further characterizing the cellular components of the EMH. Positive myeloperoxidase (MPO) staining (Figure 1D) identified mature granulocytes. CD61 staining confirmed the presence of megakaryocytes (Figure 1E). Glycophorin A staining marked erythroid precursors (Figure 1F). Finally, CD34 (Figure 1G) and CD117 (Figure 1H) staining did not show an increase in blasts. 

The absence of malignant cells was confirmed by negative staining for HMB-45 (Figure 1I), SOX-10, and CK AE1/3, effectively ruling out metastatic melanoma and carcinoma. This comprehensive immunohistochemical profile supported the diagnosis of EMH.

Due to the right lung biopsy indicating EMH and concerns about the progression of the patient’s polycythemia vera to myelofibrosis, a follow-up bone marrow biopsy was performed within one week. Bone marrow findings revealed no increase in blasts, moderate reticulin fibrosis (World Health Organization (WHO) Grade 2), and adequate iron storage. Concurrent blood flow cytometry detected 1.5% myeloid blasts, and next-generation sequencing (NGS) identified mutations in JAK2, SRSF2, and TET2. These results, plus the splenomegaly, were consistent with the patient’s history of polycythemia vera, and supported the final diagnosis of EMH. The patient’s complete blood count (CBC) remained stable, and his chronic pneumonia improved without specific treatment. Over the following weeks, the patient experienced improvements in breathing, appetite, and weight. This diagnosis guided the decision to continue conservative management. His treatment regimen for polycythemia vera was kept unchanged, consisting of low-dose aspirin, hydroxyurea, and periodic phlebotomy, with monthly CBC monitoring to track disease progression. However, three months after the diagnosis of EMH from the lung biopsy, his polycythemia vera progressed to the blast phase, with the blast count increasing to 20%. Given his advanced age and limited treatment options, management was continued with hydroxyurea and allopurinol.

## Discussion

Normal versus pathologic EMH

While EMH is a normal part of fetal development, in adults it is generally considered pathologic, except in small pools found in the spleen, liver, gut, gingiva, or lungs [5]. In healthy adults, hematopoietic progenitor cells that circulate in the circulatory or lymphatic systems stay near the bone marrow by binding their receptors to CXCR4 and CXCL12 [1].

EMH in the pleura

Primarily, EMH occurs in the spleen or liver [5]. Pleural EMH is much less common and can present variably as pleural effusion, thickening, or both, often without symptoms [6,7]. Due to its rarity and variable presentation, pleural EMH in patients with a history of malignancy may be mistaken for metastasis [5].

Diagnostic challenges

Nonetheless, there are sometimes findings on imaging that can help differentiate between EMH and malignant tumors. Older EMH lesions tend to accumulate hemosiderin, which causes them to appear hypointense on T2* or diffusion-weighted imaging on MRI, as opposed to malignancy, which would appear hyperintense [8]. On a CT, new lesions are hypoattenuating, while old lesions have attenuation similar to skeletal muscles [8]. The presence of fat deposits in EMH can also potentially be seen on imaging and be used to differentiate it from malignant tumors [8]. In this specific case, there was no evidence of these findings inconsistent with malignancy in the radiology report for the pleural enhancement caused by EMH.

Association with melanoma

Also, because the patient had a history of melanoma and was rapidly losing weight, metastatic melanoma was high on the differential. However, a diagnosis of melanoma cannot preclude the presence of EMH, and melanoma has been shown to be associated with EMH even in patients without a history of blood disorders [9]. In a recent study examining the mechanism of EMH associated with melanoma in a mouse model, it was found that melanoma-associated EMH is not caused by a defective bone marrow. Instead, the melanoma response mimics that of inflammation, leading EMH to accommodate the need for increased immune cell production [10]. Interestingly, there have been reports of a possible association between EMH and choroidal melanoma specifically, which is the melanoma type our patient had [9].

Association with polycythemia vera and myelofibrosis

Furthermore, the history of polycythemia vera pointed to EMH [11,12]. The patient’s advanced age, defined as over the age of 60, and marked splenomegaly were all risk factors for the progression of polycythemia vera to post-polycythemia vera myelofibrosis, and post-polycythemia vera myelofibrosis can be associated with compensatory EMH [13,14]. The mechanism by which this EMH occurs is thought to be due to changes to the bone marrow microenvironment that cause hematopoietic progenitors to circulate inappropriately rather than appropriately returning to the bone marrow [15]. While non-hepatosplenic EMH is not very common, splenomegaly caused by EMH, along with weight loss, is considered an important clinical feature of post-polycythemia vera EMH and is therefore a finding that can put EMH higher on the differential in a case such as this one [15,16]. In this case, after the histopathological diagnosis of EMH at an abnormal location and given the absence of significant symptoms directly attributable to EMH, a conservative treatment approach focusing on polycythemia vera management was maintained [14].

## Conclusions

In conclusion, even in patients with a history of myeloproliferative disorders, diagnosing EMH can be challenging, especially when it presents in unusual locations with nonspecific symptoms. A history of cancer further complicates this process, increasing the risk of misdiagnosis as malignancy. Accurate differentiation of EMH from malignancy is crucial to prevent unnecessary interventions, guide appropriate treatment, and improve patient outcomes. Integrating radiologic imaging and pathological evaluation plays a key role in resolving this diagnostic dilemma, underscoring the broader importance of recognizing EMH in clinical practice. 
